# Intramural Esophageal Dissection: A Rare Cause of Acute Chest Pain after Percutaneous Coronary Intervention

**Published:** 2019-07

**Authors:** Seifollah Abdi, Mohammad Reza Baianati, Mahmood Momtahen, Bahram Mohebbi

**Affiliations:** 1 *Cardiovascular Intervention Research Center, Rajaie Cardiovascular, Medical and Research Center, Iran University of Medical Sciences, Tehran, Iran.*; 2 *Madaen Hospital, Tehran, Iran.*

**Keywords:** *Percutaneous coronary intervention*, *Esophagus*, *Dissection*, *Chest pain*

## Abstract

Intramural esophageal dissection is a condition that typically presents with chest pains and may be associated with hematemesis, odynophagia, and hematemesis. The role of antiplatelet/anticoagulant agents in the development of intramural esophageal hematoma is controversial. The management of intramural esophageal dissection is generally conservative with low mortality and morbidity. The case described here is a 66-year-old woman who presented with chest pains, odynophagia, and dysphagia 1 month after percutaneous coronary intervention while taking ASA (80 mg daily) and clopidogrel (75 mg daily) for dual antiplatelet therapy. The patient was diagnosed as intramural esophageal dissection and underwent successful conservative medical management. The relative contribution of dual antiplatelet therapy with ASA and clopidogrel after percutaneous coronary intervention in this case is, albeit uncertain, a possibility.

## Introduction

Intramural esophageal dissection (IED) is a rare condition that typically presents with retrosternal chest pains and may be associated with hematemesis, odynophagia, and dysphagia.^1^^-^^3^ It can masquerade as cardiac or thoracic emergencies such as acute myocardial infarction and aortic dissection. IED is mostly reported in elderly patients, especially women.^1^^-^^3^ The role of antiplatelet/anticoagulant agents in the development of IED is controversial.^4^ We report a case of IED presenting 1 month after percutaneous coronary intervention (PCI) while receiving dual antiplatelet therapy, including ASA and clopidogrel. 

## Case Report

A 66-year-old woman with a history of controlled hypertension presented with IED 1 month after PCI and the stenting of the left anterior descending artery and the right coronary artery while taking ASA (80 mg daily) and clopidogrel (75 mg daily). The patient presented to the emergency department with severe sudden-onset retrosternal chest pains with radiation to the interscapular region associated with odynophagia and dysphagia. She had 1 episode of hematemesis shortly after presentation. She had neither a history of hard food or foreign body ingestion nor trauma before presentation. 

Electrocardiography (ECG) and laboratory studies were obtained. The ECG showed a normal sinus rhythm with no significant abnormality. She was hemodynamically stable at presentation with a heart rate of 80 beats per minute, blood pressure of 140/90 mmHg, and a respiratory rate of 18 breaths per minute. No fever was detected. In the physical examination, the patient was conscious and complained of retrosternal chest pains. The cardiac examination was unremarkable, and the lung examination revealed reduced respiratory sounds in the base of the right lung with dullness on percussion in that region. The abdominal examination demonstrated no tenderness or organomegaly. The examination of the peripheral and carotid pulses indicated no abnormality. The laboratory data showed a hemoglobin level of 11.6 g/dL.

**Figure 1:**
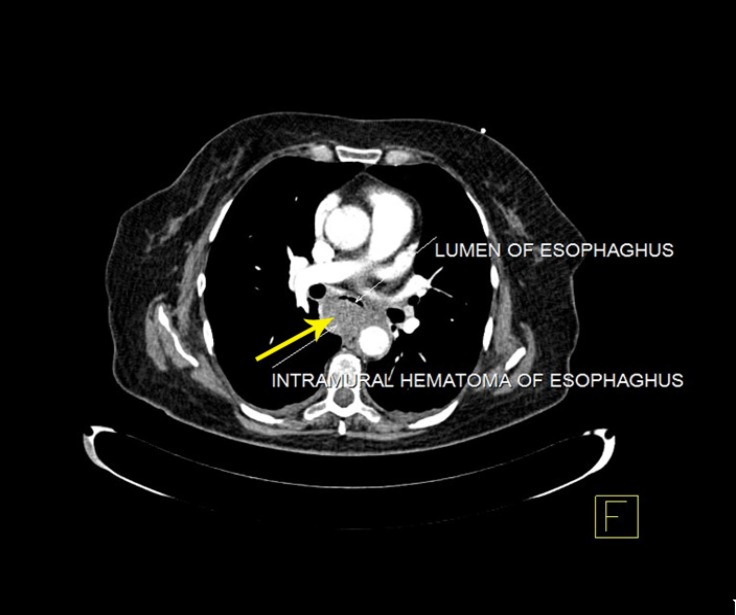
Computed tomography angiography, showing a large mass-like area along the posterior aspect of the thoracic esophagus (arrow)

**Figure 2:**
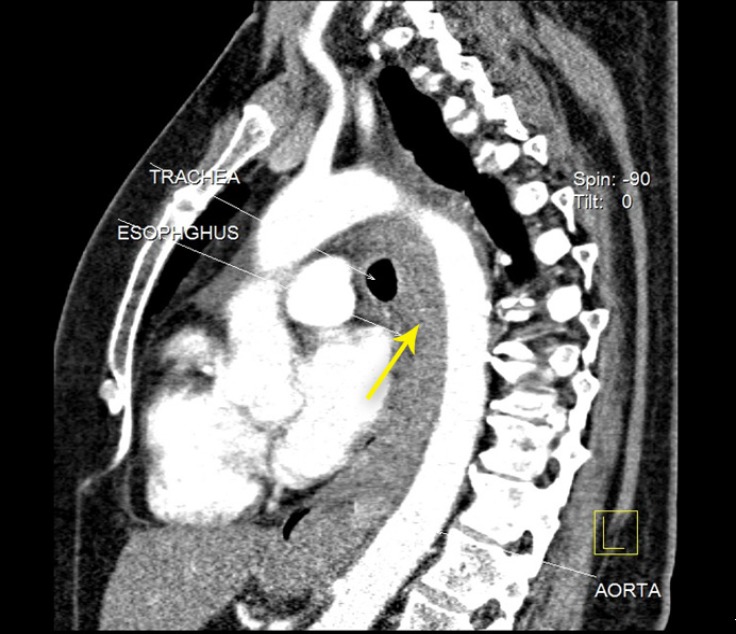
Computed tomography angiography, showing a large mass-like area along the posterior aspect of the thoracic esophagus (arrow)

Given the possibility of acute aortic syndromes, computed tomography angiography (CTA) was performed. The CTA demonstrated no significant abnormalities in the aortic or peripheral arteries, but it illustrated a large mass-like area along the posterior aspect of the thoracic esophagus and mild bilateral pleural effusion (more at the right side) (Figure 1 and Figure 2). 

The patient was transferred to the intensive care unit (ICU), where an urgent gastroenterology consult was requested and, consequently, an upper gastrointestinal endoscopic examination was performed. It depicted a long segment of esophageal denudation with no active bleeding. No bleeding site was reported in the stomach or the duodenum. 

ASA and clopidogrel were discontinued, and the patient underwent conservative management in light of the cessation of active bleeding.

Laboratory tests were repeated while the patient was in the ICU 8 hours after presentation; they were within the normal limits except for hemoglobin (8.9 g/dL). A chest roentgenogram revealed mild right-sided pleural effusion. Other laboratory tests including coagulation tests, troponin, platelet, and white blood cell counts were within the normal limits. Serial hemoglobin levels were checked, and the patient was observed for recurrent bleeding during hospitalization. She had melena on the first day of hospitalization. She was retained in the ICU for 2 days and then transferred to the ward. There were no signs of recurrent bleeding and no further reductions in the hemoglobin level. Accordingly, ASA (80 mg daily) and clopidogrel (75 mg daily) were initiated. 

The patient was discharged home after 4 days of uncomplicated hospitalization on the mentioned antiplatelet regimen. CTA was done 2 months after discharge and showed the complete resolution of the intramural esophageal hematoma.

## Discussion

 The first case of IED was reported in 1986.^5^ IED is a rare but important cause of chest pains. Clinical manifestations are retrosternal chest pains, odynophagia, hematemesis, and dysphagia. The classic triad of chest pains, dysphagia, and hematemesis is present in only 35% of cases.^6^ Physical examinations are unreliable and have limited value in the setting of IED. 

IED is a rare entity and can masquerade as other life-threatening emergencies such as acute coronary syndromes, thoracic aorta dissection, pulmonary embolism, and peptic ulcer.

The most common location of IED is the distal third of the esophagus in 83% of cases.^7^^, ^^8^ Spontaneous IED is mostly seen in middle-aged or old women.^9^ Secondary IED may be caused by endotracheal intubation, foreign body ingestion, pill-induced esophageal injury, vomiting, and endoscopic procedures.^10^^, ^^11^

The role of antiplatelet/anticoagulant therapy and coagulopathy in the development of IED is controversial. Some authors have posited that coagulation disorders, anticoagulants, or antiplatelet agents are not causative but rather aggravating factors.^12^^, ^^13^ Antiplatelet drugs are postulated to extend the hematoma rather than causing submucosal bleeding.^14^ On the other hand, some authors have considered coagulopathy or antiplatelet/anticoagulant agents as the causative factor for the intramural hematoma of the esophagus.^15^ Moreover, the in-hospital course can be complicated by myocardial infarction, which can be diagnosed by ECG and serial troponin measurements.^16^

In our patient, dual antiplatelet therapy after PCI may have had a role in the development of IED. The best initial imaging study for the diagnosis of IED is CT scan, although direct visualization with upper endoscopy is a valuable option.^17^^, ^^18^ Endoscopic ultrasound or magnetic resonance imaging may be beneficial in establishing the diagnosis.^19^^, ^^20^ The clinical course of IED is usually benign. Supportive care for IED leads to full resolution in 80% of patients.^21^

## Conclusion

Intramural esophageal dissection is a rare condition that presents with chest pains, dysphagia, hematemesis, and odynophagia. The relative contribution of dual antiplatelet therapy after PCI in the patient presented here is, although uncertain, a possibility. The morbidity and mortality related to this condition, if managed supportively, are low.

## Notes:

***This paper should be cited as: ***Abdi S, Baianati MR, Momtahen M, Mohebbi B. Intramural Esophageal Dissection: A Rare Cause of Acute Chest Pain after Percutaneous Coronary Intervention. J Teh Univ Heart Ctr 2019;14(3):138-140.
